# Formation of the Structure, Properties, and Corrosion Resistance of Zirconium Alloy Under Three-Roll Skew Rolling Conditions

**DOI:** 10.3390/ma18245578

**Published:** 2025-12-11

**Authors:** Anna Kawałek, Alexandr Arbuz, Kirill Ozhmegov, Irina Volokitina, Andrey Volokitin, Nikita Lutchenko, Fedor Popov

**Affiliations:** 1 Metal Forming Department, Czestochowa University of Technology, ul. J.H. Dabrowskiego 69, 42-201 Czestochowa, Poland; anna.kawalek@pcz.pl (A.K.); kvozhmegov@wp.pl (K.O.); 2 Core Facilities and HPC, AEO Nazarbayev University, 53 Kabanbay Batyr Ave., Astana 010000, Kazakhstan; nikita.lutchenko@nu.edu.kz (N.L.);; 3 Metal Forming Department, Karaganda Industrial University, 30 Republic Ave., Temirtau 101400, Kazakhstan; i.volokitina@tttu.edu.kz (I.V.);

**Keywords:** zirconium-based alloy, corrosion behavior, corrosion testing, radial shear rolling of bars

## Abstract

Zirconium and its alloys are widely used in nuclear power engineering due to their favorable physical and mechanical properties and their low thermal-neutron absorption cross-section. Their high corrosion resistance in aqueous and steam environments at elevated temperatures is essential for the reliable operation of fuel assemblies and is associated with the formation of a stable, compact ZrO_2_ oxide layer. However, under reactor conditions, the presence of hydrogen, iodine and other fission products can reduce corrosion resistance, making detailed corrosion assessment necessary. Manufacturing technology, alongside alloy composition, also plays a decisive role in determining corrosion behavior. This study presents corrosion test results for a Zr-1%Nb alloy processed under thermomechanical conditions corresponding to rolling in a special type of three-roll skew rolling–Radial-Shear Rolling (RSR). The applied rolling technology ensured the formation of a pronounced ultrafine-grained (UFG) structure in the near-surface layers, with an average grain size below 0.6 µm. EBSD and TEM observations revealed a largely equiaxed microstructure with refined grains and increased grain boundary density. The corrosion testing was performed in high-temperature steam vessels at 400 °C and 10.3 MPa for 72, 336, 720 and 1440 h. The results demonstrate that RSR processing is an efficient alternative to conventional multi-pass normal bar rolling with vacuum heat treatments, allowing a significant reduction in processing steps and eliminating the need for expensive tooling and intermediate thermal or chemical treatments. Bars manufactured using this method meet the ASTM B351 requirements. The specific weight gain did not exceed 22 mg/dm^2^ after 72 h and 34.5 mg/dm^2^ after 336 h. After 1440 h, the samples exhibited a continuous, uniform dark-grey oxide layer with an average thickness below 5.3 µm.

## 1. Introduction

Structural components made of zirconium alloys are used in the design of fuel assemblies for nuclear reactors [1,2,3,4]. These materials are susceptible to corrosion, both on the external surface, which is exposed to water or a mixture of water vapor, and on the internal surface, where they interact with moisture, hydrogen, fluorine and radioactive isotopes of iodine, cesium, cadmium and other elements [5,6,7,8]. Due to the presence of hydrogen, iodine and fission products in the reactor environment, which significantly intensify corrosion processes, accurate assessment of corrosion resistance and control of oxidation and hydration mechanisms of zirconium alloys are key elements in ensuring their long-term and safe operation [9,10,11,12]. The corrosion resistance is determined by the alloy composition and the manufacturing technology of the components [13,14,15,16].

**Figure 1:**
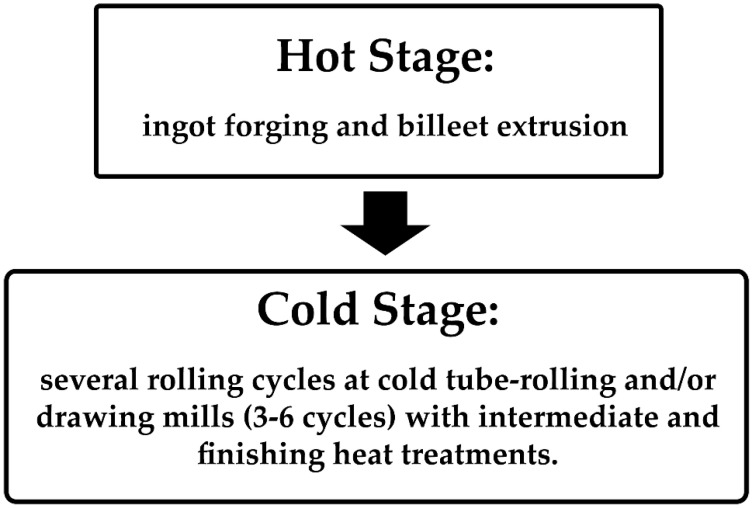
The main stages of the technology of manufacturing products from zirconium-based alloys.

The production of components made of zirconium alloys is characterized by two main processing stages (Figure 1): the hot stage (ingot forging and billet extrusion) and the cold stage (pilger rolling on cold tube-rolling mills and drawing with intermediate heat treatments). The attainment of the required mechanical properties and geometrical dimensions is primarily determined during these two stages.

This technological chain ensures reliable compliance with the requirements of the technical standards ASTM B351 [17] and ASTM B353 [18]. However, the conventional manufacturing process for zirconium-based alloy products remains highly labor-intensive due to the large number of plastic deformation steps and intermediate thermal–mechanical treatments. Moreover, the overall process yield is relatively low because of significant technological losses [19,20,21].

The improvement of manufacturing technologies for zirconium alloy components is continuously pursued with the aim of increasing fuel burnup and reducing production costs. One of the promising directions in this technological development is the application of severe plastic deformation (SPD) processes.

An analysis of the deformation schemes used in the fabrication of zirconium alloy products (tubes and bars) has shown that incorporating severe plastic deformation (SPD) into the hot-working stage is impractical due to its low efficiency at elevated temperatures (~600–1050 °C), which promote intensive grain growth. The application of SPD at the final stage of the technological cycle is also associated with high metal losses caused by deviations in geometrical dimensions and surface quality. The most suitable stage for introducing SPD processes into the technological chain of zirconium alloy fabrication is the processing of hot-extruded (or hot-rolled) sleeves and bars. Implementing SPD at this stage enables an improvement in structural homogeneity and a refinement of the grain size compared to the conventional microstructural condition of semi-finished products.

An analysis of existing publications on the application of severe plastic deformation (SPD) to zirconium alloys has demonstrated the feasibility of using the radial-shear rolling technology [22,23,24,25], which shows strong potential for industrial implementation.

According to the authors, the advantages of applying the radial-shear rolling process include:

1. It is characterized by high efficiency in terms of material and time costs when producing experimental and pilot batches from expensive metals (including zirconium-based alloys), while ensuring the formation of an ultrafine-grained (UFG) structure.
2. Easy integration into the existing manufacturing process, as it does not require the production of expensive tooling.
3. It allows for a reduction of the overall technological cycle by increasing the processability of the metal, thereby eliminating intermediate thermal and chemical treatment steps.

This study reports the results on the corrosion behavior of Zr-1%Nb alloy bars subjected to radial-shear rolling.

## 2. Materials and Methods

### 2.1. Experimental Part

The study used a E110 alloy (Zr-1%Nb) rod with an ultrafine-grained (UFG) structure obtained by the RSR method under warm deformation conditions. The billet was produced within the framework of previous work focused on investigating the possibilities of forming a UFG structure in zirconium alloys using intensive plastic deformation methods.

The concept of this work is based on the possibility of improving both the techno-economic efficiency of production and the quality characteristics of Zr-1%Nb alloy bars. The techno-economic indicators can be enhanced by shortening the costly multi-pass pilger rolling process, which involves multiple intermediate and final vacuum heat treatments. As an alternative, the use of the radial-shear rolling (RSR) technology under warm deformation conditions (T ≈ 0.3 T_m_) is proposed. The quality indicators—such as mechanical properties and corrosion resistance—are expected to improve due to the formation of an ultrafine-grained (UFG) structure.

These studies investigated the processing of initial rods of zirconium-based alloys. The results demonstrated that the combination of compressive and tensile stresses, generating a vortex metal flow, enables effective refinement of the initial coarse-grained structure to a submicron, ultrafine-grained state [24].

The material used in this study was processed by the RSR method in two stages on different mills: from an initial diameter of 37 mm to an intermediate billet size of 20 mm, and then from 20 mm to the final diameter of 13 mm.

Rolling was performed under maximum permissible mechanical and technological conditions with single reductions of 1.5–3 mm per pass. This processing route allowed for a high degree of accumulated deformation in a single heating cycle, ensuring intensive development of the UFG structure throughout the entire cross-section of the billet. A complete description of the rolling process and its parameters is provided in the publication [24].

Transmission electron microscopy (TEM) confirmed the formation of two types of structures: elongated grains oriented along the rod axis in the central region, and equiaxed UFG structure in the peripheral zones. This demonstrates the feasibility of obtaining a UFG structure in a zirconium rod in a single rolling stage without intermediate recrystallization treatment. At the same time, the presence of a pronounced structural gradient necessitated a more detailed study of the thickness, uniformity, and evolution of the UFG layer. Electron Backscatter Diffraction (EBSD) mapping was conducted, collecting 14 scans along the radial direction from the surface toward the center of the sample with a spacing of 0.5 mm between the scanned areas.

**Figure 2:**
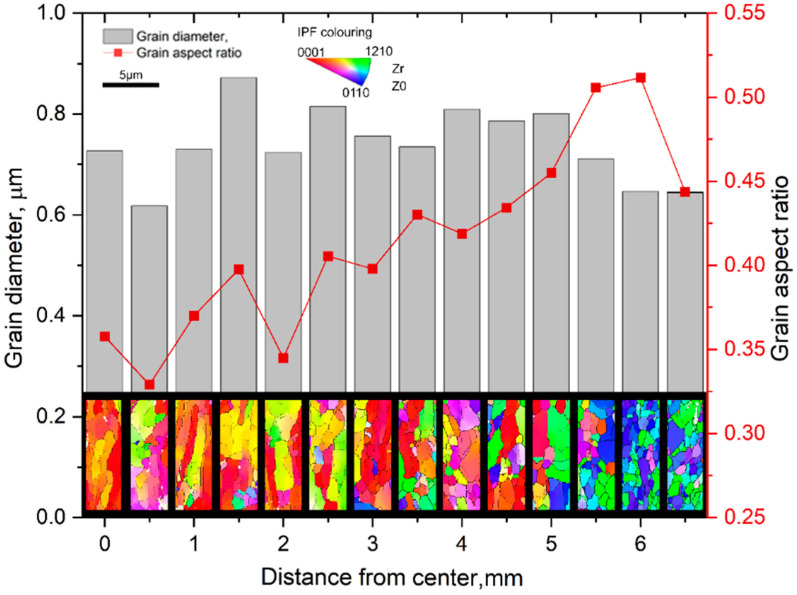
EBSD map of the sample rolled to 13 mm.

The results of EBSD mapping (Figure 2) showed that the grain refinement process begins in the surface layers at the early stages of deformation and gradually progresses toward the interior as the diameter decreases. In the final 13 mm diameter rod, a homogeneous ultrafine-grained (UFG) structure is formed with a pronounced radial gradient: the UFG layer reaches a thickness of approximately 2 mm, and the average grain size in this zone is 0.3–0.6 µm. The central region retains an elongated subgrain structure with a grain size around 0.8 µm [26].

### 2.2. Sample Preparation

**Figure 3:**
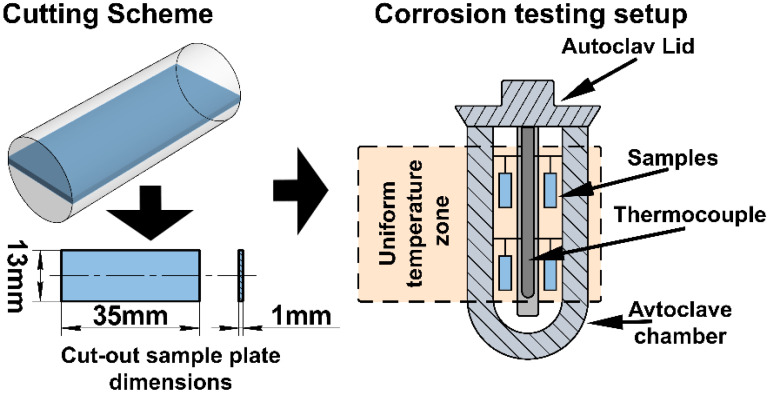
Scheme of sample preparation and corrosion testing setup.

Sample preparation was performed using a Brilliant-220 precision cutting machine (QATM, Mammelzen, Germany) with intensive water cooling, a feed rate of 5 µm/s, and a rotation speed of 700 rpm, using coarse-grained abrasive discs to minimize thermo-mechanical damage to the microstructure. From the final 13 mm diameter, 35 mm long rod, three 1 mm thick plate samples, oriented along the rod axis, were cut for corrosion testing. The sample cutting scheme and corrosion testing setup are shown in Figure 3.

### 2.3. Corrosion Test

Corrosion tests of the samples were carried out in an autoclave unit under high-temperature steam at T = 400 °C and a pressure of 10.3 MPa for 72 h and 336 h. The testing conditions correspond to the requirements of standards ASTM B351 [17] and ASTM B353 [18]. The arrangement of the samples in the autoclave working zone is shown in Figure 2. Temperature control in the working zone was performed using two chromel–copel thermocouples, while pressure was monitored by a manometer. The temperature gradient within the working zone did not exceed ±2 °C.

After 72, 336, 720, and 1440 h of exposure, the samples were removed from the autoclave for visual inspection and determination of specific weight gain. The tests with exposure times exceeding 336 h were conducted to evaluate the corrosion resistance during prolonged operation and to identify, if present, the conditions leading to the degradation of the dark-gray oxide coating-the so-called transition period of oxidation.

In accordance with the relevant standards, corrosion resistance is evaluated by determining the weight gain of samples after exposure in the autoclave. In addition to weight gain measurements, corrosion resistance is also assessed through visual inspection. A metal that exhibits satisfactory corrosion resistance after testing is characterized by a uniform dark-gray oxide film. In contrast, corrosion-resistant failure is indicated by a surface color ranging from grayish and light-gray to bright white, resembling chalk. Localized corrosion damage appears as white spots or patches. The white oxide film is structurally loose, has poor thermal conductivity, and causes overheating of the underlying metal, thereby accelerating corrosion. Moreover, this white film tends to spall off and contaminate the primary circuit with zirconium oxide particles.

Corrosion tests are carried out under conditions relevant to actual operation in order to justify the use of a material or a component made from that material. Traditionally, for the needs of the nuclear industry, corrosion testing of structural elements of fuel assemblies is performed in autoclave systems according to the regimes specified in ASTM and other regulatory documents. At the same time, other methods for evaluating corrosion resistance also exist [27], including express methods using electrochemical equipment (potentiometric techniques) [15,28]. However, the authors do not possess data confirming the interchangeability of these two corrosion testing approaches. For this reason, the authors employ the traditional methodology for conducting corrosion studies.

### 2.4. FIB Preparation

**Figure 4:**
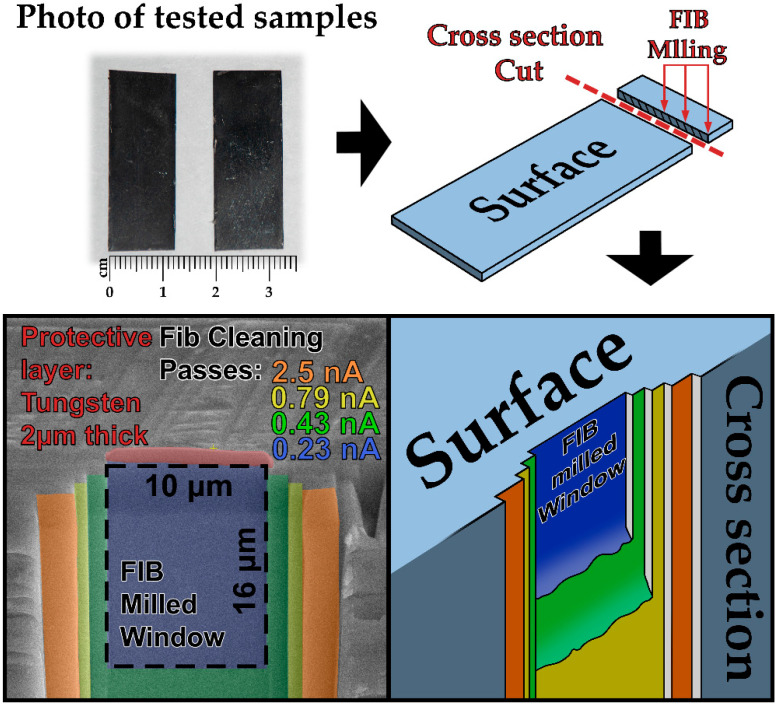
Sample preparation process scheme of the analyzed site.

After the corrosion tests, the plates were not subjected to any additional mechanical or chemical treatment, such as polishing, grinding, etching. To preserve and minimize any possible damage to the oxide film formed during the tests, the sample was sectioned using a diamond cutting disc on a Low-Speed Saw (Buehler, Switzerland) at minimal speed. To assess the thickness of the oxide layer in the cross-section, a ~4 mm-wide slice was cut from the sample (Figure 4). The cross-section of the plate was then ground with abrasive papers of progressively finer grit sizes (P800 → P1200 → P2500) to remove any residual cutting marks.

To accurately determine the thickness and structure of the oxide layer in the cross-section, Focused Ion Beam (FIB) milling was employed. This method enabled the creation of a precision cross-section within a well-defined area without disturbing the structure of the oxide layer or the underlying metal substrate [29,30]. As a result, a high-quality image of the oxide/metal interface was obtained, allowing for an accurate morphological assessment of the layer.

The study was conducted using a FIB/SEM Helios-5CX scanning electron microscope (Thermo Fisher, Eindhoven, the Netherlands) equipped with an EDAX Octane Elite Super EDS system (EDAX, Mahwah, NJ, USA). This method allows for precise site-specific milling of small areas to measure the oxide layer thickness and perform elemental analysis via EDS. On each of the three samples, three clean areas were milled with the ion beam in characteristic regions: the surface, the center (rod axis), and an intermediate area. The cutting scheme and the locations of the milled areas are shown in Figure 4.

Before milling, a protective tungsten layer was deposited to prevent the oxide layer from direct exposure to the ion beam. The layer was 2 µm thick, 3 µm wide, and 10 µm long (corresponding to the width of the intended ion-milled area). FIB milling of the area was performed in several consecutive cleaning passes, removing 0.5–0.6 µm of material per pass, at a beam voltage of 30 kV with gradual reduction in both beam current and milled area.

At the initial stage, a relatively high beam current was used to rapidly remove a large volume of material. Subsequently, the current was gradually reduced to enhance the resolution and surface quality. This approach allowed for an optimal balance between speed, precision, and minimal damage to material. The ion beam currents for each cleaning cut were: 2.5 nA → 0.79 nA → 0.43 nA → 0.23 nA. FIB milling was performed with a 1.5° tilt during the first two steps to compensate for energy loss and beam defocusing while removing a large material volume in depth. For the subsequent steps, an overtilt of 0.5° was used to achieve maximum perpendicularity of the cut relative to the sample surface. The final milled area measured 10 µm × 16 µm. The stepwise milling of the sample cross-section is shown in Figure 4.

## 3. Results and Discussion

### Corrosion Surface Analysis

The oxidation behavior of the Zr-1%Nb alloy samples demonstrates the following kinetics of oxide layer growth. Steam transfers water molecules to the surface of the oxide film, where they are adsorbed and, upon capturing electrons, form oxygen and hydrogen ions. The oxygen ions migrate through the thickness of the oxide film, reach the metal surface, and form ZrO_2_ molecules, thereby contributing to the growth of the oxide layer. The film consists mainly of monoclinic zirconium dioxide (ZrO_2_) and is characterized by high density and strong adhesion to the metal surface.

**Figure 5:**
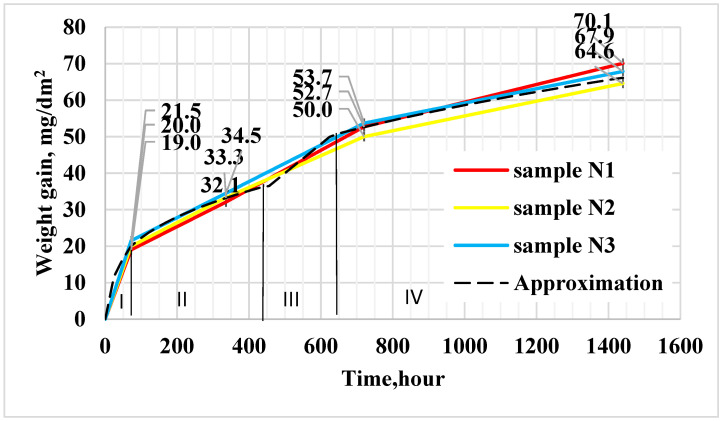
Results of specific weight gain measurements as a function of exposure time obtained after corrosion tests of Zr-1%Nb alloy samples.

After the corrosion tests (72, 336, 720, 1440 h), the samples were examined for surface appearance, and the specific weight gain was analyzed. According to the visual inspection results, the samples exhibited a continuous, uniform dark-gray oxide film, which is considered a satisfactory condition. No white oxide formations were observed. The formed oxide layer effectively protects the sample material from the detrimental effects of oxidation. The results of the specific weight gain measurements are presented in Figure 5.

Results of measurements of specific weight gain depending on the holding time shown in Table 1.

The mathematical expression of the parabolic law is Δm = 2.36 t^0.5^, where Δm is the weight gain, c is the constant, and t is the time (for oxide thickness up to ≈1 µm-exposure time ≈72 h).

The kinetic equation for the cubic parabola is Δm = 4.88 t^0.33^ (for oxide thickness of approximately ≈1–2.5 µm-exposure time 72–336 h).

The kinetic equation for oxide thicknesses of 2.5–3.5 µm (exposure time 336–720 h) is Δm = 0.08 t, and for oxide thicknesses of ≈3.5–4.5 µm (exposure time 720–1440 h) the kinetic equation is Δm = 6 t^0.33^.

**Figure 6:**
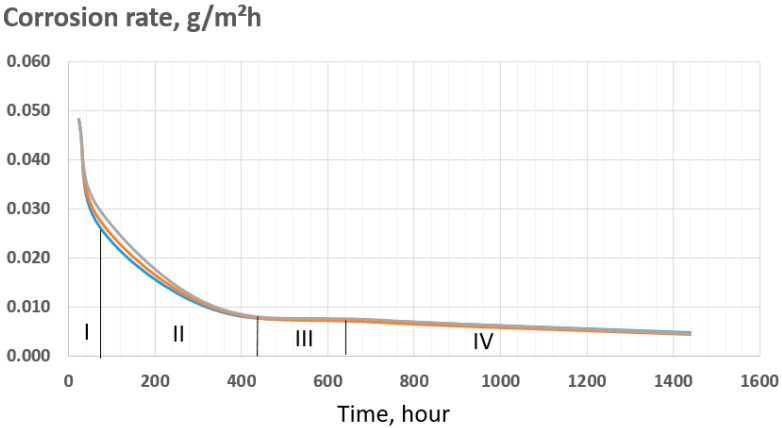
Time-dependent behavior of the corrosion rate under autoclave conditions.

For the quantitative assessment of oxidation kinetics, the corrosion rate was determined (as shown in Table 2), and a plot of the corrosion rate versus exposure time was constructed, presented in Figure 6.

Based on the analysis of specific weight gain, it can be stated that the samples successfully passed the corrosion tests at exposure durations of 72 and 336 h, remaining within the upper limit of the permissible range (not exceeding 22 mg/dm^2^ after 72 h or 38 mg/dm^2^ after 336 h).

The oxidation kinetics of zirconium and its alloys evolve over time according to different laws. The initial oxidation stage is described by a parabolic equation, which reflects the fact that the rate of oxide film growth is inversely proportional to its thickness. This behavior corresponds to the diffusion of oxygen through the zirconium dioxide lattice via anionic vacancies within the oxide crystals [12,31].

When the oxide film reaches a thickness of approximately 1 µm (after 72 h of exposure), the parabolic oxidation kinetics are replaced by a cubic law, which cannot be explained by a simple diffusion mechanism of oxygen penetration through the oxide layer.

When the oxide film reaches a thickness of about 2–3 µm (after 336–720 h of exposure), a transition phenomenon occurs, leading to a shift toward a linear oxidation law. At this stage, the film exhibits micropores of various sizes as well as longitudinal and transverse microcracks, which have been confirmed by metallographic examinations.

The linear character of the oxidation kinetics can be explained by the fact that the oxide film retains an extremely thin layer of zirconium dioxide adjacent to the metal surface, which remains free from pores and cracks. This barrier layer maintains a constant thickness, and therefore the rate of oxygen ion transport through it remains constant, resulting in linear oxidation kinetics. According to the test results, no transition period was observed; that is, the color of the oxide film did not turn gray or white.

**Figure 7:**
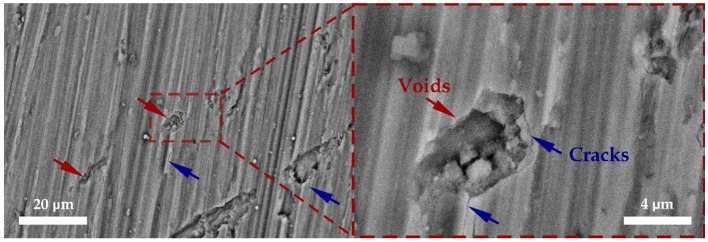
SEM images of the E110 zirconium alloy surface after corrosion testing: left—overall surface topography; right—close-up view of surface voids and cracks.

After the corrosion tests, the surface of the oxide layer was examined using scanning electron microscopy (SEM) (Figure 7). This technique enabled a detailed assessment of the oxide layer’s condition and morphology, as well as the identification of typical surface defects.

On the surface of the oxide layer, voids ranging from 5 to 50 µm are observed. Microcracks with a width of approximately 100 nm and a length of up to 10 µm extend from some of these voids. Overall, the surface exhibits a developed network of small defects characteristic of pitting corrosion.

**Figure 8:**
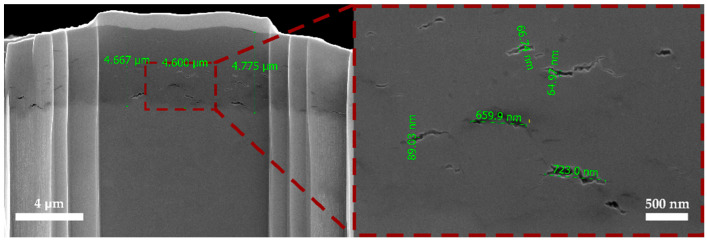
SEM image of oxide layer cross section and zoomed view of their cracks.

SEM images of the clean areas of the cross-section (Figure 8) clearly show a uniform oxide layer formed on the alloy surface, with a distinct and continuous interface with the substrate/base metal. Within the oxide layer structure, small pores and cracks with widths of 50–80 nm and lengths of 500–800 nm were observed.

The thickness of the oxide layer was measured in several zones of the cross-section, from the peripheral region with an ultrafine-grained structure to the transitional zone and the axial region with elongated grains. Measurements were taken at three points within a single FIB-milled area, and the average value was then calculated. The data for all measured oxide layer thicknesses are presented in Table 3. Similar measurements were performed on three samples. It was found that the average oxide layer thickness in the peripheral region is 5.3 µm, in the transitional zone 4.3 µm, and in the axial region 4.5 µm. These results indicate a trend of decreasing oxide layer thickness from the periphery to the axial region, which can be attributed to the structural features and varying degrees of deformation of the material in different zones [15].

Such a distribution of oxide layer thickness can be associated with microstructural inhomogeneity and differences in crystal defect density across the cross-section. In the peripheral region with an ultrafine-grained structure, the high density of grain boundaries promotes more intensive diffusion-driven oxidation, resulting in a thicker oxide film. Moving toward the axial region, where the defect density is lower, the oxide formation rate decreases, which is reflected in a reduced layer thickness.

**Figure 9:**
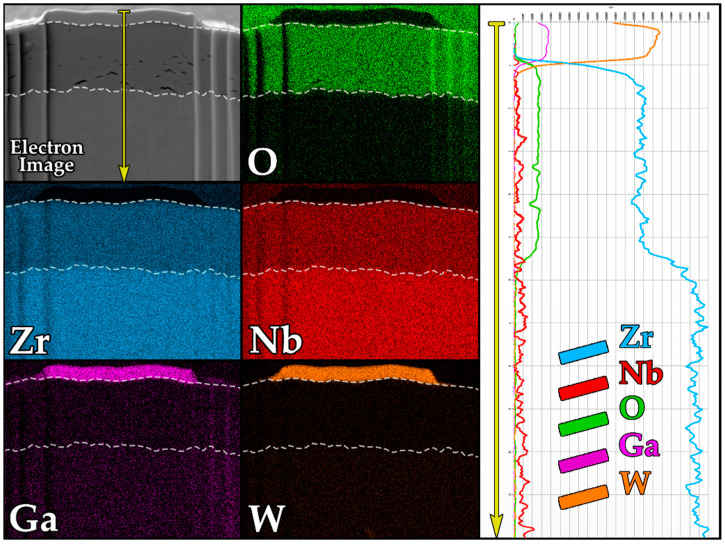
Maps and line EDS is an analysis of the cross-section of the oxide layer of the E110 alloy after corrosion tests.

Based on the EDS maps, the individual layers visible in the SEM images can be clearly differentiated by their elemental composition (Figure 9). To provide a more detailed representation of the elemental distribution across the layer thickness, a line EDS analysis was performed, traversing all identified layers.

The analysis results show the presence of a deposited tungsten layer, characterized by a high concentration of implanted gallium introduced during FIB cross-section preparation. Beneath this layer lie the oxide and metallic zirconium layers, clearly distinguished by their composition. According to the intensity profile, the base material consists predominantly of zirconium with a small amount of niobium. The oxide layer is characterized by reduced zirconium content and increased oxygen content, unambiguously indicating its identity as zirconium oxide.

## 4. Discussion

1. The significant reduction of costly technological operations, such as cold pilger rolling (up to 3–4 cycles) involving intermediate and final vacuum heat treatments, through the application of radial-shear rolling of zirconium alloy bars under warm deformation conditions enables the production of components with the required corrosion resistance. The corrosion resistance was verified for compliance with the technical requirements specified in the ASTM B351–97 standard [17].
2. Extended corrosion test exposure up to 1440 h confirmed satisfactory corrosion resistance. The resulting oxide film thickness (≈5.3 µm) is significantly below the threshold values associated with accelerated corrosion and falls within the range of typical thicknesses observed for zirconium alloys produced by traditional methods; therefore, it does not pose a risk to the operational properties of the material.
3. The observed variation in oxide layer thickness across the radial cross-section indicates a pronounced trend associated with microstructural inhomogeneity. In the peripheral zone, characterized by increased grain boundary density, the oxide film forms thicker, which is likely due to more active diffusion-driven oxidation. Towards the axial region, where defect density is lower, the oxide thickness decreases. However, establishing the exact relationship between grain boundary density and oxide layer thickness requires a separate and more detailed investigation, which will be the subject of future work.
4. In this study, no unequivocal positive effect of the UFG structure on corrosion behavior was observed. It is possible that the exposure duration was insufficient, and the effect may become apparent with longer holding times. Furthermore, the influence of the ambient atmosphere during heating for radial-shear rolling (up to T = 500 °C) cannot be ruled out, as the process eliminated several cycles of vacuum heat treatment that normally prevent zirconium interaction with the atmosphere. In this scenario, oxygen likely diffused into the bar material along grain boundaries during heating. However, it would be incorrect to definitively claim the absence of a positive effect from the UFG structure in this specific case. While the correlation between the UFG structure and corrosion behavior does not strictly align with some literature data, the significant reduction of the technological cycle must be considered. We posit that the positive effect of the UFG structure lies in the fact that, despite the significant shortening of the production cycle and the elimination of expensive operations such as pilger rolling and intermediate/final vacuum annealing, the bars processed by radial-shear rolling under warm deformation conditions successfully passed the corrosion tests.

## 5. Conclusions

Based on the test results, the following conclusions can be drawn:

1. The samples taken from rods rolled by radial-shear rolling, after corrosion tests with exposure durations of 72 h and 336 h, demonstrated a satisfactory condition in terms of appearance and weight gain.
2. The influence of the structure, including grain size, on the oxide layer’s thickness was observed. The average oxide layer thickness was 5.3 µm in the peripheral zone, 4.3 µm in the transition zone, and 4.5 µm in the central part. The more intensive oxidation in the peripheral layers is presumably due to differences in the stress state. To reduce weight gain and minimize the variation in oxide layer thickness, optimization of the final heat treatment regime can be considered to achieve a more uniform structure across the rod cross-section.
3. A corrosion-based justification has been established for applying the radial-shear rolling process to Zr-1%Nb alloy rods under warm deformation conditions as a more cost-effective alternative to the multi-pass pilger rolling process with intermediate and final vacuum heat treatments.
4. The study of the corrosion behavior kinetics of Zr-1%Nb alloy samples after radial-shear rolling will be continued for exposure times up to 3600 h.
5. No unequivocal influence of the ultrafine-grained structure on the corrosion properties was observed in the conducted tests. At the same time, the samples subjected to warm deformation via radial-shear rolling demonstrate satisfactory corrosion resistance, which is particularly important given the significant reduction of the technological cycle and the elimination of costly vacuum heat treatment and pilger rolling operations.

## Figures and Tables

**Table 1:** Results of measurements of specific weight gain depending on the holding time, obtained after corrosion tests of Zr-1%Nb alloy samples.

Exposure Time, h	Weight Gain, mg/dm^2^ Sample 1	Weight Gain, mg/dm^2^ Sample 2	Weight Gain, mg/dm^2^ Sample 3
72	19.0 ± 2	20.0 ± 2	21.5 ± 2
336	32.1 ± 3	33.3 ± 3	34.5 ± 3
720	52.7 ± 4	50.0 ± 4	53.7 ± 4
1440	70.1 ± 5	64.6 ± 5	67.9 ± 5

**Table 2:** Corrosion rate of the specimens during autoclave testing.

Exposure Time, h	Corrosion Rate, g/m^2^·h Sample 1	Corrosion Rate, g/m^2^·h Sample 2	Corrosion Rate, g/m^2^·h Sample 3
72	0.026	0.028	0.030
336	0.010	0.010	0.010
720	0.007	0.007	0.007
1440	0.005	0.004	0.005

**Table 3:** Oxide layer thickness in different areas of the sample.

№	Surface Thickness of Oxide Layer, µm	Middle Thickness of Oxide Layer, µm	Center Thickness of Oxide Layer, µm
1	5.827	4.398	4.384
2	5.558	4.384	4.492
3	5.113	3.979	4.694
4	5.679	4.263	4.721
5	5.447	4.708	4.613
6	5.571	4.357	4.91
7	4.479	4.425	4.465
8	4.937	4.209	4.087
9	5.086	4.263	4.222
**Average**	**5.299667**	**4.331778**	**4.509778**
**StdDev**	**0.40408442**	**0.1846828**	**0.2424604**

## Data Availability

The original contributions presented in this study are included in the article. Further inquiries can be directed to the corresponding author.
